# Changing epidemiology of Q fever in Germany, 1947-1999.

**DOI:** 10.3201/eid0705.010504

**Published:** 2001

**Authors:** W Hellenbrand, T Breuer, L Petersen

**Affiliations:** Robert-Koch-Institut, Berlin, Germany. HellenbrandW@rki.de

## Abstract

The epidemiology of Q fever in Germany was examined by reviewing relevant studies since 1947 and by analyzing available surveillance data since 1962. The average annual Q fever incidence nationwide from 1979 to 1989 was 0.8 per million and from 1990 to 1999, 1.4 per million. The mean annual incidence from 1979 to 1999 ranged from a minimum of 0.1 per million in several northern states to 3.1 per million in Baden-Württemberg, in the South. We identified 40 documented outbreaks since 1947; in 24 of these sheep were implicated as the source of transmission. The seasonality of community outbreaks has shifted from predominantly winter- spring to spring-summer, possibly because of changes in sheep husbandry. The location of recent outbreaks suggests that urbanization of rural areas may be contributing to the increase in Q fever. Prevention efforts should focus on reducing sheep-related exposures, particularly near urban areas.


### Changing Epidemiology of Q Fever in Germany, 1947-1999

**Wiebke Hellenbrand, Thomas Breuer, and Lyle Petersen**
Robert Koch-Institut, Berlin, Germany

The epidemiology of Q fever in Germany was examined by reviewing revelant studies since 1947 and by analyzing available surveillance data since 1962. The average annual Q fever incidence nationwide from 1979 to 1989 was 0.8 per million and from 1990 to 1999, 1.4 per million. The mean annual incidence from 1979 to 1999 ranged from a minimum of 0.1 per million in several northern states to 3.1 per million in Baden-Württemberg, in the south. We identified 40 documented outbreaks since 1947; in 24 of these, sheep were implicated as the source of transmission. The seasonality of community outbreaks has shifted from predominantly winter-spring to spring-summer, possibly because of changes in sheep husbandry. The location of recent outbreaks suggests that urbanization of rural areas may be contributing to the increase in Q fever. Prevention efforts should focus on reducing sheep-related exposures, particularly near urban areas.

Q fever is caused by the pleomorphic, obligate intracellular rickettsial agent *Coxiella burnetii*, which has an envelope similar to that of gram-negative bacteria. It is found worldwide except in New Zealand (1). Its most important reservoirs are ticks and ruminant animals such as cows, sheep, and goats (1-4). Although infection rarely causes major clinical symptoms in animals (5), it has been associated with infertility (6,7) and abortion (8,9), particularly in first-bearing newly infected parturient animals (10). Birth products from infected animals thus contain high concentrations of *C. burnetii* and can be an important source of environmental contamination (11). Transmission to humans and other animals by the aerosol route is facilitated by the tenacity of *C. burnetii*'s survival for months to years in a sporelike state on wool or fur contaminated with infected tick feces, in water, and in soil (12).

Acute Q fever in humans is characteristically a febrile, flulike illness associated with pneumonia or hepatitis (2,3,13-18). Rare complications include myocarditis, pericarditis, or meningoencephalitis (1,13,19). The death rate among persons with Q fever pneumonia is 0.5% to 1.5% (14). Up to half of patients may suffer protracted fatigue and weakness after acute disease (20,21). Rarely, more serious forms of chronic disease (most commonly endocarditis but also chronic hepatitis and vascular, osteoarticular, or pulmonary infections) may develop months to years after the acute infection (13,22,23). An increased long-term risk for arterial disease and death has also been observed (24).

Q fever was first recognized in southern Germany when several large outbreaks occurred in rural communities in 1947 to 1948 (25-28). Since then, it has been endemic in Germany. In the 1990s, several large outbreaks were recognized and investigated (5,29-36). This led us to review the epidemiology of this disease in Germany.

Address for correspondence: Wiebke Hellenbrand, Department of Infectious Diseases Epidemiology, Robert Koch-Institut, Stresemann Str. 90-102, 10963 Berlin, Germany; fax: 30-4547-3533; e-mail: HellenbrandW@rki.de

### Methods

Clinically manifest Q fever has been statutorily notifiable since 1962 in the former West Germany and since 1979 in the former German Democratic Republic (GDR, East Germany). In 1991, the two reporting systems were amalgamated. The Robert Koch Institute in Berlin receives weekly reports of the number of persons with diagnosed (laboratory-confirmed or epidemiologically linked) Q fever from local health departments through the state health authorities. Local health departments receive notification of Q fever infections from hospitals and physicians in private practice. The Federal Office of Statistics prepares annual statistical reports of notifiable diseases. We reviewed surveillance data on Q fever from humans in Germany since 1962 from these sources.

The mean annual incidence rate was calculated for the periods from 1979 to 1989 and from 1990 to 1999 by taking the mean number of persons with Q fever reported per year during each period and dividing by the 1985 and 1995 German populations, respectively. The mean annual incidence rate for the period 1979 to 1999 was calculated by taking the mean number of persons with Q fever reported per year and dividing by the 1990 German population. Incidence rates for each of the 16 German states were calculated similarly.

We reviewed veterinary surveillance data based on passive, biannual reports of the number of domestic animal herds with laboratory-confirmed Q fever (37). To obtain further information on the epidemiology of Q fever in Germany, we searched databases (MEDLINE, from 1966; EMBASE, from 1974; AGROKAT, from 1960; CAB ANIMAL, from 1972; and CABVET SCI, from 1972) and reviewed the cited literature in the retrieved relevant articles. In addition, we contacted local health departments for unpublished details on recent outbreaks.

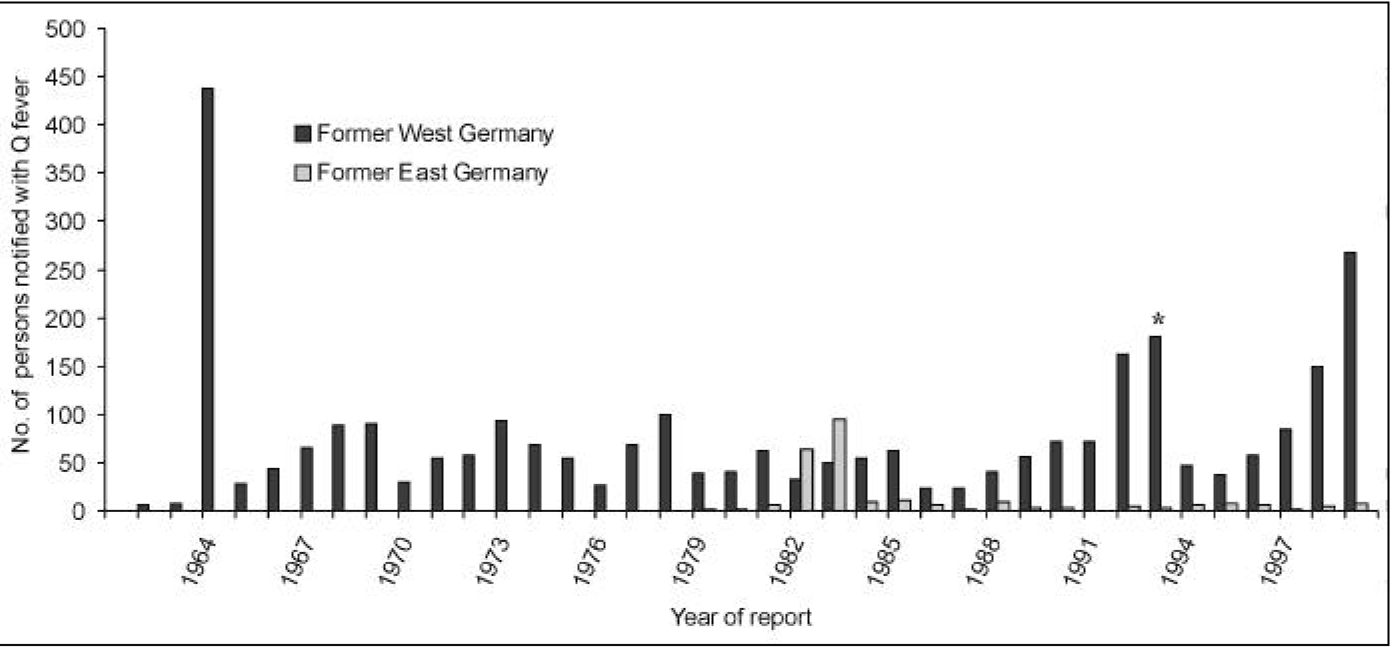

Figure 1. Number of reported cases of Q fever in Germany, 1962-1999.
*In 1993, 184 persons with Q fever were officially reported; 101 of these persons were part of the outbreak in Oberscheid, Hesse, and were only reported to the Robert Koch Institute and not to the federal Office of Statistics (36). However, a total of 97 symptomatic persons with serologically confirmed Q fever were described in a report of the outbreak in Dortmund, Northrhine-Westphalia, and 43 serologically confirmed symptomatic cases were reported in an outbreak among military recruits in Sontra, Hesse (Table 1). Thus a minimum of 94 persons with Q fever were not officially reported in 1993. (In Northrhine-Westphalia, 34 cases were reported; thus a minimum of 97 - 34 = 63 cases were not reported. In Hesse, 117 cases were reported, including 105 in conjunction with the Oberscheid outbreak [Table 1]; thus, a minimum of 43 - [117-105] = 31 cases were not reported from Hesse). Sources: (36), Robert Koch-Institute, and Federal Office of Statistics, Wiesbaden.

### Results

### Surveillance

Human Q fever surveillance data since 1962 indicate an irregular cyclic incidence pattern (Figure 1). Very few cases were reported from states constituting the former GDR (approximately one fourth of the German population), with the exception of the years 1982 and 1983, when an outbreak occurred in Thuringia (38). To consider the long-term incidence pattern between 1962 and 1999, data from West German states must be considered separately. Except for 1979 to 1991, when there is little cyclicity, the interval between the peaks is approximately 4 to 6 years. The peak in 1993 with 181 (278, if unreported cases from two outbreaks [30,35] are taken into account) and the peak in 1999 with 268 cases (West German states) were the highest since 1964, when 437 cases were reported.

The average annual incidence of Q fever in Germany from 1979 to 1999 was 1.1 per million population. From 1979 to 1989, the incidence was 0.8 per million population, and from 1990 to 1999, 1.4 per million population. The mean annual incidence rates calculated from 1979 to 1999 were generally higher in the southern German states–highest in Baden-Württemberg (4.1 per million), followed by Hesse (2.8 per million), Rhineland-Palatinate (0.9 per million), and Bavaria (0.8 per million) (Figure 2). The high average incidence observed in West Berlin (1.4 per million) is explained by an outbreak in 1992 (5,39). Had the outbreak not occurred, an incidence rate of 0.2 per million would have been observed in Berlin in this period.

### Outbreak

We documented 40 outbreaks of Q fever in humans from 1947 to 1999 (Table 1). In all but three outbreaks, the disease was confirmed in at least some of the persons affected, either serologically or by transmission from human serum or sputum to mice or guinea pigs.

Sheep were implicated in the transmission of Q fever to humans in at least 24 of the documented outbreaks (in six rural community outbreaks the source was uncertain). Exposure to products of conception was explicitly considered the potential source of infection in 11 of the sheep-associated outbreaks. In the outbreak in Dortmund, Northrhine-Westphalia, a case-control study revealed that exposure to manure contaminated with the products of conception from infected sheep was associated with Q fever (35). Infectious dust produced by shearing of sheep whose wool was presumably contaminated with infected tick feces was suspected as another possible source of transmission, since shearing and disease occurrence were temporally associated in 3 of these 11 outbreaks and as the main source in one other outbreak. In 12 outbreaks, sheep located near or migrating through inhabited areas were implicated without specification of the presumed mechanism of transmission. Dry weather or wind blowing from areas where sheep were located to inhabited areas was thought to play a contributory role in at least 14 outbreaks.

Cattle were suspected as the source of infection in six outbreaks; four were community outbreaks (40-43). In the Niederrhein outbreak in 1958 (42,43), exposure to products of conception aborted by a seropositive cow and to other seropositive cows sold at an animal fair was considered to be the source of infection (42,43). Exposure to infected cows,

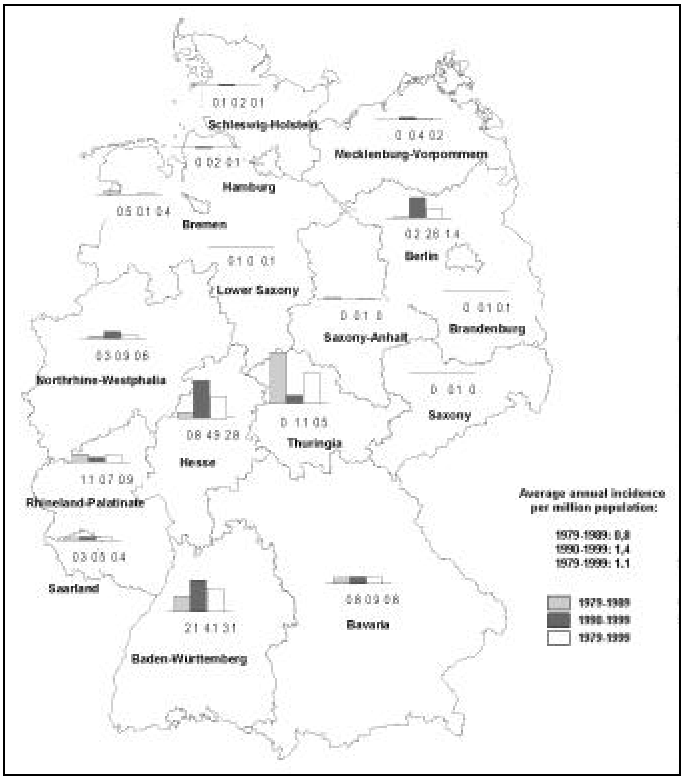

Figure 2. Mean annual Q fever incidence per million population in Germany.

some shedding *C. burnetii* in their milk, was implicated in the community outbreaks in Neulussheim, Zuzenhausen, and Schwaikheim in Baden-Württemberg (40,41). The other two cattle-associated outbreaks occurred in abattoirs (44,45). In one additional abbatoir-related outbreak, the source of infection could not be identified (46).

Two laboratory outbreaks occurred in 1947 and 1948 (26,47), but none have been reported since. Exposure to sputum of a patient who contracted Q fever in a laboratory that contained high concentrations of *C. burnetii* caused a hospital outbreak in 1948 (28).

The seasonality of community outbreaks in Germany has changed during the past decade compared to earlier years (Figure 3), with a marked decrease in winter outbreaks and an increase in summer ones. In contrast to earlier community outbreaks, which were almost exclusively rural, recent outbreaks have frequently involved people living in or near urban areas (5,30,34,35,48).

### Seroprevalence Studies

Seroprevalence studies of *C. burnetii* antibodies in the German population have not been performed recently. Of 1,611 serum samples collected from blood donors from all 16 German states from 1983 to 1986, 22% were positive for *C. burnetii* antibodies using a phase I/II immunoglobulin (Ig) G enzyme-linked immunosorbent assay (ELISA) (49). In this survey, 15% of the 205 specimens from Hesse were positive. None of 1,075 sera from blood donors from Hesse had been positive for *C. burnetii* using complement fixation (CF) (positive titer 1:10) in a 1977 survey (50). This suggests a possible increase in exposure to *C. burnetii* during this interval, although ELISA has a higher sensitivity than the CF test (1). State-specific surveillance data from Hesse also suggest an increase in disease activity during this interval: The annual number of reported human Q fever cases was 0.6 cases per year between 1962 and 1977 but averaged 5.3 cases per year from 1978 to 1986. In a seroprevalence study among German federal armed forces personnel from 1985 to 1987 (51), 22% of 1,651 blood donors had antibodies to *C. burnetii* using the same ELISA as used by Schmeer et al. (49).

### Q Fever in Animals

The extent of disease in animals over time is difficult to quantify. State veterinarians are required to report biannually the number of herds in which one or more animals have laboratory-confirmed Q fever (37). The average annual number of reported herds with Q fever was 71 between 1971 and 1979, 328 between 1980 and 1989, and 303 between 1990 and 1998. However, this does not permit inferences about the extent of infection, as neither the number of herds nor the number of animals tested is available. Furthermore, testing is not representative because the disease is generally asymptomatic in animals. Most testing occurs routinely in dairy cattle whose milk is destined for human consumption without pasteurization or in cattle destined for slaughter; otherwise, testing occurs only sporadically, as when the number of abortions increases, infertility is noted, or an outbreak of human disease is thought to be related to an animal source. In 1998, a survey requesting information on the number and results of Q fever testing in state veterinary laboratories in 13 of the 16 German states was performed (52). This showed that 7.8% of 21,191 tested cattle, 1.3% of 1,346 tested sheep, and 2.5% of 278 tested goats had evidence of *C. burnetii* infection by various tests (52). Again, the reasons for testing are not available; thus, the results cannot be considered representative of Q fever in animals in general and give only a rough indication of the extent of disease.

Seroprevalence surveys in cattle reveal that *C. burnetii* infection has been endemic in cattle in southern Germany since the 1950s; the seroprevalence reported in various surveys is generally >5% (53-55). Baden-Württemberg may be an exception (7), as a very low cut-off titer was chosen there, making comparison with other studies difficult (Table 1). Although different tests were used over time and between locations, seroprevalence in cattle in southern Germany appears to have remained fairly constant since the early 1950s. However, in other parts of Germany that have data available over time (e.g., Hesse, Northrhine Westphalia, Lower Saxony), seroprevalence was very low in the 1950s and 1960s but increased to levels comparable to those in southern Germany in the 1980s and 1990s (Table 1). In herds that had problems with infertility or abortions, seroprevalence rates were as high as 75% (6,7).

Seroprevalence of *C. burnetii* antibodies in surveys of sheep has generally been lower than in cattle (Table 2). This is likely partially due to the longer persistence of antibody titers in cattle (13). However, when surveys were performed in flocks of sheep implicated in human outbreaks, much higher seroprevalences were often found (Table 2), presumably reflecting more recent, acute infection with *C. burnetii.*

### Discussion

The number of persons reported annually with Q fever in Germany rose markedly in the 1990s. Although this could

Table 1. Seroprevalence of antibodies to *Coxiella burnetii* in cattle in Germany

| Survey location | Year(s) | No. of animals | No. of herds | Test used | Seroprevalence (%) | Seropositive herds (%) |
|---|---|---|---|---|---|---|
| Northern Germany | | | | | | |
| Schleswig-Holstein (56) | 1955-56 | 425 | 36 | Microagglutination[a] (≥1:20) | 0 | 0 |
| Lower Saxony (57) | 1970 | 400 | N/A | CF[b] (≥1:20) | 11 | N/A |
| Lower Saxony (58) | 1992-93 | 665 | 39 | CF (≥1:20)<br>CF (≥1:10) | 4.7<br>9.6 | 76.9 |
| Eastern Germany | | | | | | |
| Former East Germany (59) | 1980-89 | 95,464 | N/A | CF (≥1:20), except 913 sera with ELISA) | 8.3[c] | N/A |
| Middle/Western Germany | | | | | | |
| Hesse (60) | 1953 | 585 | 97 | Transmission from milk to guinea pigs; CF (≥1:5)[d] | 0 | 0 |
| Northrhine-Westphalia (61) | 1958-60 | 2,157 | N/A | CF (≥1:20)<br>CF (≥1:5) | 0.1<br>0.5 | N/A<br>N/A |
| Northrhine-Westphalia (62) | 1981-83 | 5,184 | 297 | CF (≥1:20)<br>CF (≥1:5) | 3.8<br>6.7 | <br>23.6 |
| Hesse (6) | 1982-83 | 3,200 | 591 | ELISA[e]<br>CF (≥1:20) | 13.4<br>6.3 | 29.6 |
| Northrhine Westphalia (63) | 1989-90 | 3,500 | 155 | ELISA<br>IFT[f] (≥1:8) | 13.3<br>12.9 | 57.4<br>53.5 |
| Southern Germany | | | | | | |
| Bavaria (53) | 1953 | 1,000 | N/A | CF (≥1:16)<br>CF (≥1:32) | 10.1<br>5.9 | N/A |
| Bavaria (64) | 1968 | 1,000 | N/A | CF (>1:20) | 8.4 | N/A |
| Baden-Württemberg (7) | 1984 | 2,109 | 125 | CF (≥1: 5) | 8.0 | 35.2 |
| Northern Bavaria (54) | 1983-84 | 3,384 | 246 | CF (>1:20) | 7.6 | 30.0 |
| Southern Bavaria (55) | 1991 | 1,095 | 21 | ELISA | 11.8 | 81.0 |

ELISA = enzyme-linked immunosorbent assay; CF = complement fixation; IFT = immunofluorescence testing; N/A = not available.
[a]The sensitivity and specificity of microagglutination are comparable to that of the ELISA (1).
[b]CF is less sensitive and less specific than ELISA, IFT, and microagglutination.
[c]17.3% among dairy cattle.
[d]According to a study by Schaal and Schäfer (62), 33% of cows seropositive at a titer of 1:10 and 65% positive at a titer of 1:20 shed *C. burnetii* in their milk.
[e]The ELISA used in Germany and in all studies listed in this table (except for [59], for which no information was provided) is based on the test developed by Schmeer et al. (49,65), which contains both phase I and phase II antigens and detects immunoglobulin (Ig) G as well as IgM-antibodies. The following antigens are included in this ELISA: Nine Mild Strain (phase II), Munich Strain (phase I and II), and Frankfurt Strain (mainly phase I). A net absorption cutoff of 0.200 read at 450 nm was used. In a large seroprevalence survey by Gouverneur (6), all of the sera testing positive by CF at a titer ≥1:20 tested positive by ELISA. However, while 13.4% of sera tested positive by ELISA, only 6.3% tested positive using CF at a titer≥1:20. At a CF titer of 1:10, only 58% of positive sera also tested positive by ELISA. However, at this titer, 8.4% of sera tested positive by CF. This is still 37% less than found using the ELISA. In a smaller study by Schmeer et al. (49), all sera testing positive at a titer ≥1:16 tested positive by ELISA.
[f]The IFT is considered the reference method of Q fever serodiagnosis (1).

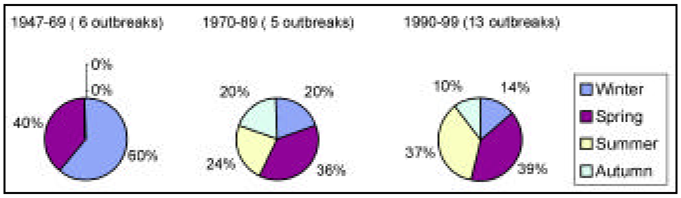

Figure 3. Seasonality of 24 sheep-associated community outbreaks in Germany, 1947-1969, 1970-1989, and 1990-1999. For each community outbreak in which sheep were implicated, the number of months' duration in each season was calculated. For each time period, the percentage of the total number of outbreak months occurring during each season was calculated. The "year-round" outbreak in Dettenhausen (Table 1) was excluded. Winter = January-March; spring = April - June; summer = July-September; autumn = October-December.

be due to enhanced awareness and better clinical recognition of the disease, the increased number of Q fever outbreaks, which are less likely to be clinically missed than sporadic cases, suggests that the increase is real. In fact, outbreaks in Baden-Württemberg, Hesse, Northrhine-Westphalia, and Berlin are responsible for most of the increase. The increased outbreak activity observed during the 1990s is reminiscent of the large outbreaks described between 1948 and 1954 (see online table of documented Q fever outbreaks in Germany from 1948 through 1999 at URL:http://www.cdc.gov/eid/vol7no5/hellebrand_table).

The seroprevalence of 22% found in blood donors and military personnel in the 1980s in Germany is probably not representative of the general population. We have no information on the proportion of rural versus urban residence for blood donors. However, a seroprevalence study of blood donors in Switzerland (using a phase II IgG immunofluorescence test with a cut-off titer of 1:20) revealed lower seroprevalences in urban areas (10.9%) as compared to rural areas (17%) (68). In addition, because soldiers often train on grounds that are grazed upon by sheep (69), their exposure to *C. burnetii* may be higher than the general population's. A seroprevalence of 4% was found in 942 blood donors in Marseille, France, in 1988 also using a phase II IgG immunofluorescence test (cut-off titer 1:25) (70). In the Netherlands, a much higher seroprevalence of 45.5% was found in sera collected from blood donors between 1968 and 1983, using a Phase II IgG immunofluorescence test (cut-off titer 1:16) (71). However, international comparisons are difficult because of varying test methods and cutoffs.

The relatively high seroprevalence of *C. burnetii* antibodies in Germans suggests that the true number of persons with Q fever exceeds the number reported. Because Q fever often presents as a flulike illness, it may not be correctly diagnosed unless there is heightened suspicion of Q fever, such as would occur during an outbreak. Even then, not all cases may be reported. In 1993, at least 94 persons with symptomatic Q fever during recognized outbreaks were not reported to the surveillance system (Figure 1).

The cyclic incidence peaks seen on the surveillance curve generally correspond to years in which one or more outbreaks were documented. Thus, the occurrence of outbreaks was largely responsible for the cyclic pattern, a finding similar to that observed in Israel (72). It is unknown whether the incidence of sporadic Q fever follows the same cyclic pattern as Q fever occurring in outbreaks.

The highest Q fever incidences in Germany were observed in Bavaria, Baden-Württemberg, Rhineland-Palatinate, Hesse, Northrhine-Westphalia, and Thuringia, with Hesse showing the greatest increase since 1990. Of the 34 community outbreaks, 33 occurred in these six states. The tick *Dermacentor marginatus*, which acts as a host for *C. burnetii* and feeds on sheep in large numbers, is endemic in the four most southern states (Bavaria, Baden-Württemberg, Rhineland-Palatinate, and Hesse) but has not been

Table 2. Seroprevalence of antibodies to *Coxiella burnetii* in sheep

| Location | Year(s) | No. of animals | No. of herds | Test used | Seroprevalence (%) | Seropositive herds (%) |
|---|---|---|---|---|---|---|
| Surveys unrelated to human outbreaks | | | | | | |
| Northrhine-Westphalia (61) | 1958-60 | 2,199 | N/A | CF (1:20) | 0 | N/A |
| Bavaria (64) | 1968 | 1,000 | N/A | CF (>1:20) | 4.5 | N/A |
| Thuringia (66) | 1980-89 | 4,337 | 17 | CF (≥1:20)<br>CF (>1:10) | 0.73<br>1.1 | 47 |
| Surveys in herds implicated in human outbreaks | | | | | | |
| Southern Germany (53) | 1953 | 31 | 1 | CF (≥16) | 32.2 | N/A |
| Rhineland-Palatinate (67) | 1974 | 265 | 1 | CF (≥1:20)<br>CF (≥1:10) | 7.9<br>12.5 | N/A |
| Rollshausen, Hesse (33) | 1996 | 20 | 1 | ELISA | 75.0 | N/A |
| Giessen, Hesse (31) | 1997 | 100 | 1 | ELISA | ~50 | N/A |
| Dortmund, Northrhine-Westphalia (35) | 1999 | 100 | 1 | ELISA | 57.0 | N/A |

N/A = not available; CF = complement fixation; ELISA = enzyme-linked immunosorbent assay.

found in the more northern states of Northrhine-Westphalia or Thuringia (59, 73-75). Excreta from infected ticks persist in animal fur as a highly infectious dust, permitting aerosol transmission within the flock as well as to humans (73). This can occur through direct or indirect contact or through shearing, as observed in the outbreaks in Baden-Württemberg in 1998 and 1999 (see online table). *C. burnetii* has also been isolated from the tick *Ixodes ricinus* in Baden (40), in Northrhine-Westphalia (61), in Hesse (69), and in Thuringia (38). A role for *I. ricinus* in the transmission cycle of *C. burnetii* in areas where *D. marginatus* is not endemic but Q fever incidence is relatively high, such as Northrhine-Westphalia and Thuringia, is plausible, although evidence is lacking thus far. A tick-independent cycle of *C. burnetii* has been observed in cattle (55).

In addition to infectious dust from tick excreta, contaminated birth products are an important source of human infection (2,12,13,76). Terhaag (77) first noted the temporal association of Q fever outbreaks with outdoor lambing in southern Germany. Exposure to infectious products of conception was implicated in 10 sheep-associated community outbreaks and one cattle-associated community outbreak in this study.

Cattle were implicated in only four community and three abattoir outbreaks, none of which occurred recently. Although the increase in seroprevalence in cattle in more northern parts of Germany has been accompanied by an increase in reported Q fever outbreaks in Hesse and Northrhine-Westphalia (but not Lower Saxony or Schleswig-Holstein), cattle were never implicated as a possible source of the outbreaks. This is likely because cattle do not migrate over large distances, they graze on pastures close to inhabited areas less frequently, and they are not shorn. Moreover, calving normally occurs indoors under more controlled conditions.

From 1947 to 1969, epidemic Q fever activity occurred mainly during winter and spring (Figure 3). From 1970 to 1989, outbreaks occurred less commonly during winter, and started to occur during summer and fall as well. Finally, since 1990, outbreak activity has decreased further during winter and increased during summer (Figure 3). The change in seasonality coincides with changes in sheep husbandry. The nomadic form of sheep husbandry practiced mainly in southern parts of Germany has become less common. In former West Germany, nomadic herds gradually decreased from 1,178 in 1968 (30% of sheep) to 917 in 1988 (27% of sheep) (78). By 1994 this had decreased further to 702 (18% of sheep, reunified Germany) (79). Limited data from the late 1940s reveal that 50% of sheep farming was nomadic in Bavaria, 55% in Baden, and 88% in Württemberg (80). In 1994, this had decreased to 28% in Bavaria and 56% in (now unified) Baden-Württemberg (79). Because winter lambing (80-82), with either spring (80,81) or winter (82) shearing, is practiced in this form of sheep husbandry, the decrease in nomadic sheep farming in Germany may be related to the decrease in outbreaks during winter.

Spring lambing and shearing are most common in other forms of sheep farming, which have increased proportionately in Germany (82). Lambing at other times of the year, however, is possible and is being increasingly encouraged to enable year-round provision of the market with fresh lamb meat (83). Increased lambing or shearing during the warmer, drier seasons could increase the risk of aerogenous spread of *C. burnetii* from birth products or wool to humans and may thus be one possible explanation for the recent seasonal shift as well as for the increase in Q fever outbreaks in the 1990s.

In addition, outbreaks have increasingly affected people living in or close to urban areas (30,34,35,48). This suggests that increased exposure of susceptible humans to sheep through urbanization into traditionally rural areas may be another factor contributing to the observed increase in outbreaks. The use of sheep for landscaping purposes on recreational and park lands has also increased in importance since the 1970s (79,84).

Taken together, the presented data suggest that conditions for the transmission of *C. burnetii* to humans mainly from sheep have become more favorable in recent years, leading to increased outbreak activity in Germany. Therefore, stricter implementation of preventive measures is essential.

Apart from attempting to prevent the disease in animals as far as possible (e.g., isolation of animals that abort, examination of aborted lambs [69,85]), preventive measures must aim at preventing human contact with potentially infectious dust in animal fur and with infectious products of conception. In areas endemic for *D. marginatus*, control of transmission from ticks to animals must be achieved by rigorous treatment of sheep with acaricides (86-88). Since 1981, acarcide treatment of sheep has been recommended in areas where *D. marginatus* is endemic (86). However, such treatment may not protect for the entire tick season (86), so avoiding close contact between tick-infested sheep and susceptible persons (particularly during shearing) remains important. Rigorous disinfection after lambing or calving, including adequate heat or chemical treatment of manure before its use as fertilizer as well as removal of birth products through a licensed institution (89-91), is essential to prevent transmission.

Finally, investigation of vaccination of sheep against *C. burnetii* as a preventive measure in Germany may be warranted, particularly in light of a recently developed chloroform-methanol residue vaccine with fewer side effects (13,92,93) and long-lasting antibody induction in sheep (94). Studies in cattle suggest that this could reduce both the degree and proportion of infectious placentas (7,54). Unfortunately, no Q fever vaccine is currently licensed for use in animals in Germany.

### Conclusion

Human disease caused by *C. burnetii* appears to have increased during the past decade in Germany. Q fever is endemic in cattle throughout Germany and in sheep at least in southern, eastern, and western parts of the country. Sheep have most often been implicated in transmission of the disease to humans. Urbanization into rural areas; increased grazing of sheep on recreational and park lands, leading to increased opportunity for contact between susceptible persons and infected animals; and changes in sheep husbandry may have contributed to the observed increase in outbreaks. Thus, awareness of this disease as a threat to human health and compliance with preventive and control measures must be improved among farmers, veterinarians, and the public.

### Acknowledgments

We thank Drs. Wolf, Dittmeier, Dreweck, Zöllner, and Pfaff for providing us with the results of Q fever outbreak investigations performed in 1999.

Dr. Hellenbrand is a research assistant at the Infectious Diseases Epidemiology Unit, with research interests in Q fever, disease surveillance, and vaccine-preventable diseases.

### References

1. Fournier P-E, Marrie TJ, Raoult D. Diagnosis of Q fever. J Clin Microbiol 1998;36:1823-34.
2. Marrie TJ. *Coxiella burnetii*. In: Mandell GL, Bennett JE, Dolin R, editors. Principles and practice of infectious diseases. Vol 2. New York: Churchill Livingstone; 1995. p. 1727-35.
3. Reimer LG. Q Fever. Clin Microbiol Rev 1993;6:193-8.
4. Werth D, Schmeer N, Müller H-P, Karo M, Krauss H. Nachweis von Antikörpern gegen *Chlamydia psittaci* und *Coxiella burnetii* bei Hunden und Katzen: Vergleich zwischen Enzymimmuntest; Immunperoxidase-Technik, Komplementbindungsreaktion und Agargelpräzipitationstest. J Vet Med 1987;B34:165-76.
5. Schneider T, Jahn H-U, Steinhoff D, Guschoreck H-M, Liesenfeld O, Mäter-Böhm H, et al. Q-Fieber-Epidemie in Berlin. Dtsch Med Wochenschr 1993;118:689-95.
6. Gouverneur K, Schmeer N, Krauss H. Zur Epidemiologie des Q-Fiebers in Hessen: Untersuchungen mit dem Enzymimmuntest (ELISA) und der Komplementbindungsreaktion (KBR). Berliner Münchener Tierärztliche Wochenschrift 1984;97:437-41.
7. Woernle H, Müller K. Q-Fieber beim Rind: Vorkommen, Bekämpfung mit Hilfe der Impfung und/oder antibiotischen Behandlung. Tierarztl Umschau 1986;41:201-12.
8. Plagemann O. Die häufigsten infektiösen Abortursachen beim Schaf in Nordbayern unter besonderer Berücksichtigung der Chlamydien-und Salmonelleninfektionen. Tierarztl Prax 1989;17:145-8.
9. Heil-Franke G, Plagemann O, Singer H. Virologische und bakteriologische Untersuchungsergebnisse von abortierten Rinderfeten aus Nordbayern. Tierarztl Umschau 1993;48:16.
10. Sanford SE, Josephson GKA, MacDonald A. *Coxiella burnetii* (Q fever) abortion storms in goat herds after attendance at an annual fair. Can Vet J 1994;35:376-8.
11. Welsh HH, Lennette EH, Abinanti FR, Winn JF. Air-borne transmission of Q fever: the role of parturition in the generation of infective aerosols. Ann NY Acad Sci 1957;70:528-40.
12. Dedié K, Bockemühl J, Kühn H, Volkmer K-J, Weinke T. Bakterielle Zoonosen bei Tier und Mensch. Epidemiologie, Pathologie, Klinik, Diagnostik und Bekämpfung. Stuttgart, Germany: Ferdinand Enke Verlag; 1993.
13. Maurin M, Raoult D. Q fever. Clin Microbiol Rev 1999;12:518-53.
14. Marrie TJ, Raoult D. Q fever--a review and issues for the next century. Int J Antimicrob Agents 1997;8:145-61.
15. Dupuis G, Peter O, Pedroni D, Petite J. Clinical aspects observed during an epidemic of 415 cases of Q fever. Schweiz Med Wochenschr 1985;115:814-8.
16. Mege JL, Maurin M, Capo C, Raoult D. *Coxiella burnetii*: the "query" fever bacterium: a model of immune subversion by a strictly intracellular microorganism. FEMS Microbiol Rev 1997;19:209-17.
17. Sawyer LA, Fischbein DB, McDade JE. Q fever: current concepts. Rev Infect Dis 1987;9:935-46.
18. Klug J, Maenicke P. Q-Fieber--eine meldepflichtige Erkrankung. Z Ärztl Fortbild 1985;79:119-20.
19. Levy P-Y, Carrieri P, Raoult D. *Coxiella burnetii* pericarditis: report of 15 cases and review. Clin Infect Dis 1999;29:393-7.
20. Marmion BP, Shannon M, Maddocks I, Storm P, Pentilla A. Protracted debility and fatigue after acute Q-fever [letter]. Lancet 1996;347:977-8.
21. Ayres JG, Smith EG, Flint N. Protracted fatigue and debility after acute Q fever [letter]. Lancet 1996;347:978-9.
22. Raoult D, Marrie T. Q Fever. Clin Infect Dis 1995;20:489-96.
23. Edlinger EA. Chronic Q fever. Zentralblatt fur Bakteriologie und Hygiene 1987;267 A:51-6.
24. Lovey P-Y, Morabia A, Bleed D, Péter O, Dupuis JP. Long term vascular complications of *Coxiella burnetii* infection in Switzerland: cohort study. BMJ 1999;319:284-6.10
25. Heni E, Germer WD. Q(eensland)-Fieber in Deutschland. Dtsch Med Wochenschr 1948;73:472-6.
26. Kikuth W, Bock M. Twenty-three cases of laboratory infection with Q fever. Med Klin 1949;44:1056-60.
27. Freygang F. Klinische, epidemiologische und serologische Beobachtungen bei Q-Fieber 1948/49 in Nord-Württemberg. Dtsch Med Wochenschr 1949;48:1457-63.
28. Siegert R, Simrock U, Stroeder U. Über einen epidemischen Ausbruch von Q-Fieber in einem Krankenhaus. Z Parasitol 1950;2:1-40.
29. Gesundheitsamt Ebersberg. Bericht über die Q-Fieber-Erkrankungen im Landkreis Ebersberg, 1999. Ebersberg: Gesundheitsamt Ebersberg; 2000.
30. Gesundheitsamt Freiburg, Landesgesundheitsamt Baden-Württemberg, Robert Koch-Institut. Ergebnisse einer Fall-Kontroll-Studie zum Q-Fieber in Freiburg 1998. Freiburg, Germany: Gesundheitsamt Freiburg; 1998.
31. Gesundheitsamt Giessen. Bericht über einen Q-Fieber-Ausbruch auf einer Lehr- und Forschungsstation der Universität Giessen. Giessen, Germany: Gesundheitsamt Giessen; 1997.
32. Landratsamt Miltenberg–Gesundheitsamt. Q-Fieber-Kleinepidemie im Landkreis Miltenberg. Miltenberg, Germany: Landratsamt Miltenberg-Gesundheitsamt; 2000.
33. Lyytikäinen O, Ziese T, Schwartländer B, Matzdorff P, Kuhnhen C, Jäger C, et al. An outbreak of sheep-associated Q fever in a rural community in Germany. Eur J Epidemiol 1998;14:193-9.
34. Kröner B. Q-Fieber–auch in Großstädten eine Gefahr. Deutsches Arzteblatt 1995;92:378-81.
35. Reintjes R, Hellenbrand W, Düsterhaus A. Q-Fieber-Ausbruch in Dortmund im Sommer 1999. Gesundh-Wes 2000;62:1-6.
36. Robert Koch-Institut. Q-Fieber-Epidemien in den Jahren 1992 und 1993. Epidemiologisches Bulletin 1994;1:4-5.
37. Federal Ministry of Nutrition Agriculture and Forestry. Q fever surveillance in domestic animals. Bonn: Federal Ministry of Nutrition, Agriculture and Forestry; 1999.
38. Kramer M. Epizootologisch–epidemiologische Untersuchungsprogramme von potentiellen Naturherdinfektionen am Beispiel des Q-Fiebers im Bezirk Suhl [dissertation]. Leipzig, Germany: Karl-Marx-University; 1990.
39. Mölle G, Hentschke J, Laiblin C. Diagnostische Maßnahmen anläßlich einer Q-Fieber-Endemie in einem berliner Schafbestand. J Vet Med 1995;B42:405-13.
40. Hengel R, Kausche GA, Sheris E. Über zwei dörfliche Q-Fieberepidemien in Baden. Dtsch Med Wochenschr 1950;45:1505-7.
41. Caesar O. Eine ausgedehnte Q-Fieber-Epidemie im Frühjahr 1950 in Nordwürttemberg. Medizinische Monatsschrift (Stuttgart) 1950;4:837-45.
42. Trüb PGC, Boese W, Posch J. Die Q-Fieber-Epidemie am Niederrhein 1958, Land Nordrhein-Westfalen. Archiv fur Hygiene und Baketeriologie 1960;144:48-73.
43. Boese W, Trüb CLP, Posch J. Ergebnisse der serologischen Untersuchungen bei der Q-Fieber-Epidemie 1958 am linken Niederrhein, Land Nordrhein-Westfalen. Zentralblatt fur Bakteriologie 1960;179:325-35.
44. Mayer H. Beobachtungen zu einer Q-Fieber-Schlachthausepidemie in Württemberg. Dtsch Med Wochenschr 1949;48:1476-7.
45. Weise H-J. Epidemiologie des Q-Fiebers beim Menschen in der Bundesrepublik Deutschland. Bundesgesundheitsbl 1971; 14:71-5.
46. Liebermeister K. Schlachthofepidemien durch Q-Fieber. Monatsheft fur Tierheilkunde 1950;2:237-42.
47. Nauck EG, Weyer F. Laboratoriumsinfektionen bei Q-Fieber. Dtsch Med Wochenschr 1949;74:198-202.
48. Schulze K, Schwalen A, Klein RM, Thomas L, Leschke M, Strauer BE. Eine Q-Fieber-Pneumonie-Epidemie in Düsseldorf. Pneumonologie 1996;50:469-73.

49. Schmeer N, Krauss H, Werth D, Schiefer HG. Serodiagnosis of Q fever by enzyme-linked immunosorbent assay (ELISA). Zentralblatt fur Bakteriologie Hyg 1987;267:57-63.
50. Schmatz H-D, Schmatz S, Krauss H, Weber A, Brunner H. Seroepidemiologische Untersuchungen zum Vorkommen von Antikörpern gegen Rickettsien beim Menschen in der Bundesrepublik Deutschland. Immunitat und Infektion 1977;4:163-6.
51. Werth D, Lampadius E, Krauss H. Seroprävalenz von Antikörpern gegen *Coxiella burnetii* bei Angehörigen der Bundeswehr (Blutspender und Krankenhauspatienten). Wehrmedizinische Monatsschrift 1991;8:369-71.
52. Hartung M. Bericht über die epidemiologische Situation der Zoonosen in Deutschland für 1998. Übersicht über die Meldungen der Bundesländer. Berlin: Bundesinstitut für gesundheitlichen Verbraucherschutz und Veterinärmedizin; 1999.
53. Germer WD, Glokner B. Epidemiologie des Q-Fiebers in Hessen: Untersuchungen mit dem Enzymimmuntest (ELISA) und der Komplementbindungsreaktion (KBR). Berl Munch Tierarztl Wochenshr 1953;97:437-41.
54. Roth CD, Bauer K. Untersuchungen zur Verbreitung des Q-Fiebers bei Rindern in Nordbayern und zu Maßnahmen zur Bekämpfung unter besonderer Berücksichtigung der Impfung. Tierarztliche Umschau 1986;41:197-201.
55. Rehácek J, Krauss H, Kocianová E, Kovacova E, Hinterberger G, Hanák P, et al. Studies of the prevalence of *Coxiella burnetii*, the agent of Q fever, in the foothills of the southern Bavarian Forest, Germany. Zentralblatt fur Bakteriologie 1993;278:132-8.
56. Wegener KH. Das Q-Fieber und seine milchhygienische Bedeutung. Kiel Milchw Forschungsber 1957;9:509-35.
57. Neumann W. Serologische Untersuchungen über das Vorkommen von Q-Fieber-Antikörpern bei Rindern im Weser-Ems-Gebiet. Tierarztliche Umschau 1971;26:384-7.
58. Wittenbrink MM, Gefaller S, Failing K, Bisping W. Einfluss von Bestands- und Tierfaktoren auf den Nachweis komplementbindender Antikörper gegen *Coxiella burnetii* beim Rind. Berl Münch Tierarztl Wochenschr 1994;107:185-91.
59. Kramer M. Zum Vorkommen, zur Verbreitung und zur Epidemiologie des Q-Fiebers in den neuen Ländern der Bundesrepublik Deutschland. Tierarztliche Umschau 1991;46:411-16.
60. Schoop G. Das Q-Fieber. Übersicht über den Stand der Forschung und Untersuchungen über das Vorkommen in Südhessen. Monatsheft für Tierheilkunde 1953;5:93-111.
61. Schaaf J. Query-Fieber des Rindes. Monatsheft für Tierheilkunde 1961;13:1-18.
62. Schaal EH, Schäfer J. Zur Verbreitung des Q-Fiebers in einheimischen Rinderbeständen. Dtsch Tierarztl Wochenschr 1984;91:52-6.
63. Klemt C, Krauss H. Zur Epidemiologie des Q-Fiebers: Vorkommen von Antikörpern gegen *Coxiella burnetii* beim Rind im Regierungsbezirk Arnsberg, Nordrhein/Westfalen (1989/90). Tierarztliche Umschau 1991;46:520-4.
64. Schliesser T, Schmidt U. Zur Verbreitung und Bedeutung des Q-Fiebers bei Schaf und Rind. Zentralbl Veterinarmed 1970;17:238-42.
65. Schmeer N, Krauss H, Wilske B. Untersuchungen zur Serodiagnose des Q-Fiebers beim Menschen -Nachweis von nicht-komplementbindenden IgM-Antikörpern im Enzyme-linked Immunosorbent Assay (ELISA). Immunitat und Infektion 1984;12:245-51.
66. Lange S, Klaus G. Seroepidemiologische Untersuchungen zum Nachweis von Q-Fieber bei Schafen in Mittel-Thüringen. Berl Munch Tierarztl Wochenschr 1992;105:333-5.
67. Schaal E, Goetz W. Über Q-Fieber-Infektionen und deren Ursache unter der Bevölkerung des Raumes Simmerath/Eifel aus tierärztlicher Sicht. Dtsch Tierarztl Wochenschr 1974;81:477-500.
68. Dupuis G, Péter O, Mottiez M-C, Vouilloz M. Séro-prévalence de la fièvre Q humaine en Suisse. Schweiz Med Wochenschr 1986;116:494-8.
69. Thoms H-J. Epidemiologische Untersuchungen zum Vorkommen von *Coxiella burnetii* auf vier Truppenübungsplätzen der Bundeswehr in Nordrhein-Westfalen und Niedersachsen [dissertation]. Giessen, Germany: Justus-Liebig-Universität Giessen; 1996.
70. Tissot-Dupont H, Raoult D, Brouqui P, Janbon F, Peyramond D, Weiller P-J, et al. Epidemiologic features and clinical presentations of acute Q fever in hospitalized patients: 323 French cases. Am J Med 1992;93:427-34.1415306
71. Richardus JH, Donkers A, Dumas AM, Schaap GJ, Akkerman JP, Huisman J, et al. Q fever in the Netherlands: a sero-epidemiological survey among human population groups from 1968 to 1983. Epidemiol Infect 1987;98:211-9.
72. Yarrow A, Slater PE, Costin C. Q fever in Israel. Pub Health Rev 1990-91;18:129-37.
73. Liebisch A, Rahman MS. Zum Vorkommen und zur vektoriellen Bedeutung der Zecken *Dermacentor marginatus* (Sulzer, 1776) und *Dermacentor reticulatus* (Fabricius, 1794) in Deutschland. Tropenmedizin und Parasitologie 1976;27:393-404.
74. Liebisch A, Burgdorfer W, Rahman MS. Epidemiologische Untersuchungen an Schafzecken (*Dermacentor marginatus*) auf Infektionen mit Rickettsien. Dtsch Tierarztl Wochenschr 1978;85:121-6.
75. Liebisch A. Die Rolle einheimischer Zecken (Ixodidae) in der Epidemiologie des Q-Fiebers in Deutschland. Dtsch Tierarztl Wochenschr 1983;83:274-6.
76. Doerr HW, Hoferer E, Leschhorn V, Nassal J. Q-Fieber: Eine in Süddeutschland endemische Infektionskrankheit. Deutsche Medizinische Wochenschrift 1981;106:1532-5.
77. Terhaag L. Zur Epidemiologie des Q-Fiebers. Q-Fieber in der Eifel. Archiv fur Hygiene und Bakteriologie 1953;137:247-69.
78. von Korn S. Schafe in Koppel- und Hütehaltung. Stuttgart, Germany: Eugen Ulmer; 1992.
79. Vereinigung Deutscher Landesschafzuchtverbände. Schafe: Fakten, Zahlen und agrarpolitische Entscheidungen zur Schafhaltung in Deutschland. Bonn: Vereinigung Deutscher Landesschafzuchtverbände; 1995.
80. Diener HO. Süddeutsche Schäferfibel. Munich: Buchdruckerei J. Gotteswinter; 1949.
81. Doehner H. Haltung, Fütterung und Krankheiten des Schafes. Berlin: Paul Parey; 1944.
82. Behrens H, Schellje R, Waßmuth R. Lehrbuch der Schafzucht. Berlin: Paul Parey; 1983.
83. Schlolaut W. Nutzungsziel Lammfleischerzeugung, 2. Konsequenzen für Management und Zucht. Deutsche Schafzucht 1993;85:56-9.
84. Hesse Department of Nutrition Agriculture and Land Development. Schafzucht und Schäfer in Hessen. Wiesbaden, Germany: Hesse Department of Nutrition Agriculture and Land Development; 1992.
85. Kaulfuss K. Infectious abortions of sheep. Man is endangered. Deutsche Schafzucht 1997;89:406-9.
86. Liebisch A. Control of *Dermacentor marginatus* in sheep in Germany. In: Whitehead GB, Gibson JD, editors. Proceedings of the International Conference on Tick Biology and Control under the Auspices of the Tick Research Unit, Rhodes University, Grahamstown, South Africa. Grahamstown, South Africa: Rhodes University; 1981. p. 157-8.
87. Liebisch A. Das Q-Fieber als Naturherdinfektion in Süddeutschland. Bundesgesundheitsbl 1977;20:185-91.
88. Liebisch A. Ecology and distribution of Q-fever rickettsiae in Europe with special reference to Germany. Recent Advances in Acarology 1979;II:225-31.
89. Böhm R, Strauch D. Desinfektion im Stall--weniger Krankheiten, mehr Leistung. Bonn: Gesellschaft fur Druckabwicklung mbH; 1996.
90. Kazár J, Brezina R. Control of rickettsial diseases. Eur J Epidemiol 1991;7:282-6.
91. Schliesser T, Kraus H. Bekämpfung des Q-Fiebers. Tierarztliche Praxis 1982;10:11-22.7179235
92. Fries LF, Waag DM, Williams JC. Safety and immunogenicity in human volunteers of a chloroform-methanol residue vaccine for Q fever. Infect Immun 1993;61:1251-8.
93. Waag DM, England MJ, Pitt MLM. Comparative efficacy of a *Coxiella burnetii* chloroform: methanol residue (CMR) vaccine and a licensed cellular vaccine (Q-Vax) in rodents challenged by aerosol. Vaccine 1997;15:1779-83.
94. Lang GH, Prescott JF, Williams JC. Serological response in sheep vaccinated against *Coxiella burnetii* (Q fever). Can Vet J 1994;35:373-4.
